# Impact of pharmacist intervention on adherence and measurable patient outcomes among depressed patients: a randomised controlled study

**DOI:** 10.1186/s12888-015-0605-8

**Published:** 2015-09-16

**Authors:** K. Aljumah, MA Hassali

**Affiliations:** 1 Department of Pharmacy, Al-Amal Psychiatric Hospital, P.O. Box 33626, Riyadh, 11458 Saudi Arabia; 2 School of Pharmaceutical Sciences, Universiti Sains Malaysia, Penang, Malaysia

## Abstract

**Background:**

Adherence to antidepressant treatment is essential for the effective management of patients with major depressive disorder. Adherence to medication is a dynamic decision-making process, and pharmacists play an important role in improving adherence to antidepressant treatment in different settings within the healthcare system. The aim of this study was to assess whether pharmacist interventions based on shared decision making improved adherence and patient-related outcomes.

**Methods:**

This was a randomised controlled study with a 6-month follow-up. Participants were randomly allocated to two groups: 1) intervention group (IG) (usual pharmacy services plus pharmacist interventions based on shared decision making); or 2) control group (CG) (usual pharmacy services). Recruited patients fulfilled the following inclusion criteria: aged 18 to 60 years diagnosed with a major depressive disorder, and no history of psychosis or bipolar disorders. A research assistant blinded to the group allocations collected all data.

**Results:**

Two hundred and thirty-nine patients met the inclusion criteria and were randomised to the IG (*n* = 119) or CG (*n* = 120). Nineteen patients dropped out of the study during the follow-up phase. After 6 months, patients in the IG had significantly more favorable medication adherence, treatment satisfaction, general overuse beliefs, and specific concern beliefs. However, the groups did not differ in severitye of depression or health-related quality of life after 6 months.

**Conclusions:**

Our findings emphasise the important role of pharmacists in providing direct patient care in regular pharmacy practice to improve adherence to medications and other patient-reported outcomes.

**Trial registration:**

ISRCTN34879893, Date assigned: 30/12/2014

## Background

Medication adherence is a dynamic behaviour that changes over time with changing beliefs and attitudes towards prescribed medications, and it reflects a patient’s effort to optimally manage health, symptoms, and physical function [1]. Adherence to antidepressant treatment is essential for the effective management of major depressive disorder (MDD) [2]. Lack of adherence affects a large proportion of patients, with up to 52 % of patients discontinuing medication after 3 months [3]. Recent data from Saudi Arabia have shown that 53 % of patients have low adherence to antidepressant medication [4].

Adherence to medication is part of a decision-making process. Patients actively make decisions about their medications after weighing their reservations against the perceived benefits [5]. For each medication, patients usually consider their respective beliefs about medication-specific concerns and beliefs about the need for medications, as well as other general beliefs [6]. Patients’ authority and their participation in the decision-making process have increased in the last two decades [7, 8]. Several terms have arisen to describe patient involvement, including ‘informed decision making’ , ‘concordance’ , and ‘evidence-based patient choice’ [9]. Furthermore, progressive concepts have developed, such as shared decision making (SDM). SDM can be defined as ‘a two-way exchange of information, consultation and decision making, where deliberation and decisions are made by both the healthcare professional and the patient’ [9, 10].

A systematic review has shown that there is a positive relationship between patient participation in the decision-making process and outcomes, satisfaction, and improved patient self-esteem, alongside decreased treatment discontinuation rates [11, 12]. Similarly, positive associations have been found between SDM involving depressed patients and patient satisfaction [13]. Additionally, evidence supports the use of decision aids to improve deliberative treatment decisions during the SDM process [14].

Depressed patients have demonstrated an interest in increasing their knowledge about treatment options and participating in treatment decisions [14, 15]. In contrast, psychiatrists have shown poor patient involvement when making treatment decisions [16]. Data from Saudi Arabia showed that the rate of involvement of depressed patients in SDM was 66 %, when measured by observing patient involvement on the decision-making scale (OPTION scale). This increased to 79 % when patients used decision aids [15].

The literature suggests that a patient’s active participation in decision making regarding treatment for depression will result in improved adherence, satisfaction, and improved clinical outcomes. Furthermore, evidence from a systematic review supports the role of pharmacists in providing various interventions to improve medication adherence in antidepressant treatment in different settings [4].

The aim of this study was to evaluate the effectiveness of SDM-based pharmacist intervention for improving adherence and patient outcomes, compared with usual care in patients diagnosed with MDD.

## Methods

This study followed the Medical Research Council guidelines for evaluating complex interventions [17]. We conducted a prospective randomised controlled study with a 6-month follow-up, beginning by randomly grouping participants into either: 1) the intervention group (IG; usual pharmacy services plus pharmacist intervention based on SDM); or 2) the control group (CG; usual pharmacy services without SDM-based pharmacist intervention). Patients in the control group received usual care and standard communication regarding their medication when they visited the pharmacy to collect their antidepressants, without any communication aimed specifically at increasing patients’ involvement, such as in SDM. Any inquiries addressed to the pharmacist were answered according to routine practice in the pharmacy.

### SDM intervention

During the intervention, pharmacists followed the SDM competency framework, which was designed specifically for depressed patients, to ensure all aspects of SDM were implemented for each patient [18]. Before the SDM session started, the research team distributed a decision aid to patients in the IG. This was developed and validated by Aljumah and colleagues [15, 19] and is specifically designed for Arabic-speaking patients [19]. The intervention focused on enhancing patients’ involvement in decision making by assessing their beliefs and knowledge about antidepressants. The average duration of the first SDM session (baseline) was 15 min, and the second session (final session) lasted 10 min (at 3-month follow-up).

### Setting

The study took place between February 2014 and July 2014 in Riyadh, the capital city of Saudi Arabia. This city has a total population of more than 5,000,000 and one psychiatric hospital (Al-Amal Hospital; total of 500 beds) is the main provider of psychiatric care for the entire population.

### Patient populations

Recruited patients fulfilled all of the following inclusion criteria

#### Inclusion criteria

- Aged 18 to 60 years;
- Newly diagnosed with an MDD, according to the criteria of the Diagnostic and Statistical Manual of Mental Disorders, 4th Ed (DSM-IV; 1994);
- No history of psychosis or bipolar disorders;
- No drug or dependency history;
- No cognitive impairment that may hinder the assessment.

Patients were excluded from further analysis for the purpose of this study if they met the following criterion:

#### Exclusion criteria

- No response at any level to the antidepressant within 8 weeks of recruitment.

Patients who met the inclusion criteria were briefed about the aims and objectives of the study and invited to participate. Written consent was sought within 24 h of the recruitment visit.

### Data collection

Baseline visits took place in the outpatient clinic, and assessments were conducted by independent raters (two trained nurses) who were blinded to patients’ group allocation. This was meant to limit potential observer bias or conscious deception, and reduce the influence of the placebo effect. At baseline, the raters recorded data pertaining to patients’ socio-demographic characteristics, adherence, beliefs, satisfaction with their depression treatment, severity of depression, health-related quality of life, and the quality of patients’ involvement in SDM during clinical interviews. The same measures were administered at the 3- and 6-month follow-up (with the exception of patient involvement in SDM, which was not measured at 6 months). Pharmacists and psychiatrists were not blinded to the patients’ group allocation.

### Randomisation

Study participants were individually randomised to one of two parallel groups with an allocation ratio of 1:1 using a computer-generated list. The computer-generated allocation was done by a research assistant with no clinical involvement in the trial. Pharmacists and psychiatrists were not blinded to the patients’ group allocation but the research assistant who collected all data was blinded to group allocation.

### Measurements and scales

#### Assessment of adherence

Direct and indirect measures can be used to evaluate patients’ medication adherence. In this study, we used an indirect method, the Morisky Medication Adherence Scale (MMAS). The MMAS is a well-validated instrument [20–23], with good reliability and an available Arabic version [24]. The MMAS, which is a useful screening technique for antidepressant medications [25], consists of eight items addressing specific medication-taking behaviours and adherence. MMAS scores range from 0 to 8, with higher scores representing better adherence. A score of less than 6 is considered to indicate poor adherence, while a score of 6 or more is considered to indicate good adherence. Written permission to use the questionnaire was obtained from its authors.

#### Assessment of patients’ beliefs about medicine

Patients’ medication-related beliefs were assessed using the Patients’ Beliefs about Medicine Questionnaire (BMQ; Specific and General versions). This self-report measure has proven validity, reliability, and psychometric capability for both general medical patients and depressed patients [3, 6, 26]. The BMQ-Specific contains two parts: ‘Specific Necessity’ , which evaluates patients’ views about the necessity and importance of their medication; and ‘Specific Concerns’ , which questions patients’ beliefs about the potential harm and adverse effects of their medications. Each part of the questionnaire has a potential score ranging from 5 to 25. The BMQ-General also has two parts: ‘General Harm’ , which measures beliefs that medicines in general are harmful, addictive or poisonous; and ‘General Overuse’ , which measures beliefs that medicines in general are overused by doctors [6]. Again, written permission was obtained from the original author.

#### Assessment of severity of depression

At each visit, the severity and progression of the disease was assessed using the Montgomery–Åsberg Depression Rating Scale (MADRS). The MADRS is an established scale that is widely used in clinical psychiatry studies to evaluate depression over time. The MADRS focuses more on the psychological symptoms of depression, as opposed to the somatic symptoms [27]. The MADRS comprises 10 items that cover symptoms typical of MDD, with a possible score of 0 to 6 for each item. The total score is a measure of the overall severity of the depression [28, 29]. A score of 30 or more indicates severe depression, while a score of 10 or below indicates remission [27, 28].

#### Assessment of patients’ involvement in decisions

SDM was assessed quantitatively during the baseline and 3-month visits using the Observing Patient Involvement in Decision-Making scale (OPTION) in the intervention group only. OPTION was developed to evaluate the extent to which patients are involved in the clinical decision-making process [16]. This validated scale consists of 12 rating items, each scored on a five-point Likert scale (0 to 4). A score of ‘0’ indicates that competency is not observed and scores of ‘1’ to ‘4’ represent increasing levels of achievement. An overall OPTION score is obtained by adding the scores for all 12 items together (maximum 48), and then standardising it to obtain a score between 0 and 100 [30]. Trained nurses administered the scale using videotapes of SDM interventions.

#### Assessment of health-related quality of life

Quality of life was assessed using the EQ-5D, a generic health-related quality of life (HRQoL) instrument developed by the EuroQoL group. This is a standardised instrument used to measure health outcomes, which provides a simple descriptive profile and single index value for health status that can be used in clinical studies [31]. In this study, we used the Arabic version of the EQ-5D [32]. The first part of the EQ-5D records self-reported problems in one of five domains (mobility, self-care, usual activities, pain/discomfort, and anxiety/depression), according to three levels of severity (no problems, some problems, and extreme problems). The second part records the subject’s self-assessed health on a Visual Analogue Scale (VAS) – a vertical 20 cm line on which the best and worst health states score 100 and 0, respectively [32].

#### Assessment of patient satisfaction with treatment

Patient treatment satisfaction was measured using the Arabic version of the self-report Treatment Satisfaction Questionnaire for Medication (TSQM 1.4). The TSQM has been shown to be psychometrically robust and to have proven validity and reliability with Arabic patients [33, 34]. It is a 14-item scale consisting of four dimensions, including effectiveness, side effects, convenience, and global satisfaction [35]. The patients were instructed not to respond to the questions in the side effects dimension if they were not suffering from side effects. TSQM 1.4 domain scores range from 0 to 100, with higher scores representing higher satisfaction in that domain [36, 37].

### Dropouts

Dropouts were defined as patients who did not attend the follow-up visits at 3 and 6 months, and who could not be assessed again. Patients who decided to stop their treatment without having experienced significant side effects were counted as non-adherents. All analyses were limited to patients who completed all assessments (complete-case analysis).

### Sample size and power

We needed to obtain a difference of at least 17 points in the percentage of medication intake at a confidence level of 95 % [38], allowing for a 20 % dropout rate [39] to provide 80 % power, assuming an alpha risk of 0.05 and a beta risk of <0.20. This gave us a target sample size of 195 patients to achieve adequate power.

### Data analysis

Descriptive and comparative statistics were analysed using SPSS. Demographic and clinical characteristics of the two study groups were compared at baseline using analysis of variance (continuous variables). ANCOVA analyses were conducted to assess differences in medication adherence, beliefs about depression severity, level of participation in SDM, and satisfaction between treatment groups, controlling for baseline scores. We conducted a mixed ANOVA to analyse changes over time and between groups. We conducted an independent sample t-test to compare overall mean adherence between groups.

### Ethical considerations

Approval for the study protocol was received from the Research Ethics Committee of the Ministry of Health in Saudi Arabia received before patients recruitment. Informed written consent was sought from all study participants. All participants were provided with a participant information sheet that included detailed information regarding the nature of the study, to enable informed consent. Participants were informed of their right to drop out of the trial at any stage, without any effects on their treatment or relationship with the treating team. Participants were assured about confidentiality and data protection of gathered personal information, which would only be used by the researchers in an anonymised format.

## Results

**Fig. 1:**
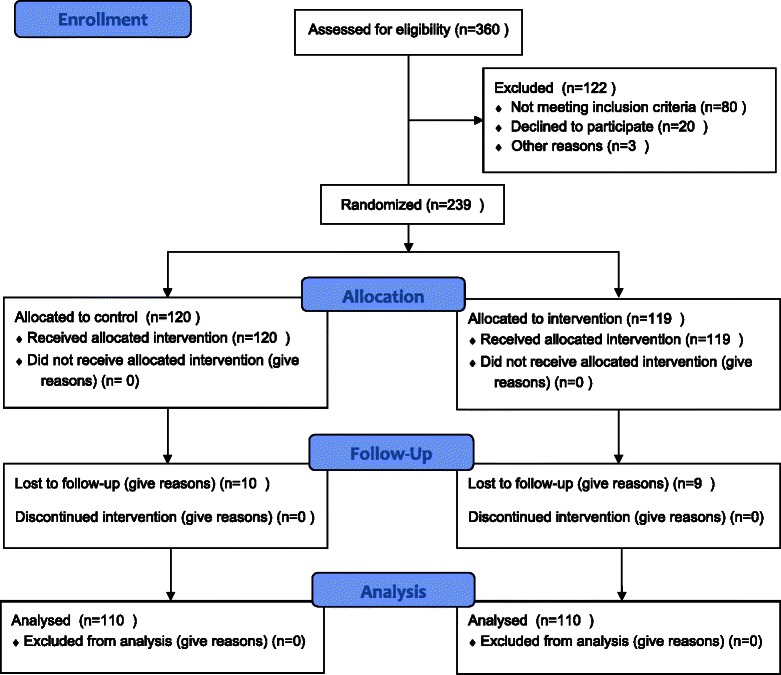
Recruitment flow diagram

Two hundred and thirty-nine patients met the inclusion criteria for this study and were randomised to the IG (*n* = 119) or the CG (*n* = 120). Of these, 19 dropped out of the study during the follow-up phase (Fig. 1) and were not included in the final analyses. In the group of drop-outs, there was a greater proportion of males and elderly patients compared with the group of patients who completed the study. No other baseline differences existed between the groups.

Table 1 shows demographic characteristics of the patients, according to group allocation. There were no statistically significant differences between the IG and CG in these variables, including age, education, and number of prescribed antipsychotics. Table 2 shows scores for clinical and patient-related outcome measures at baseline. Patients in the IG and CG reported similarly moderate severe depression, with a mean MADRS score of 22.90 (S.D. 13.27) and 21.89 (S.D. 12.89), respectively. At baseline, there were no significant differences between the groups in terms of beliefs towards antidepressant medications, treatment satisfaction scores or HLQoL.

**Table 1:** Demographic characteristics of intervention group (*n* = 110) and control group (*n* = 110) patients

	Intervention group	Control group
	No (%)	No (%)
*Sex*		
Male	49 (44.5)	51 (46.4)
Female	61 (55.5)	59 (53.6)
*Age*		
18–30 years	32 (29.1)	27 (24.5)
31–40 years	31 (28.2)	35 (31.8)
41–50 years	27 (24.5)	27 (24.5)
51–60 years	20 (18.2)	21 (19.1)
*Education level*		
Uneducated	19 (17.3)	31 (28.2)
Primary	17 (15.5)	17 (15.5)
Intermediate	21 (19.1)	23 (20.9)
Secondary	37 (33.6)	29 (26.4)
College/Bachelor	16 (14.5)	10 (9.1)
*Number of antidepressants prescribed*		
One	86 (78.2)	95 (86.4)
Two	24 (21.8)	15 (13.6)

**Table 2:** Clinical characteristics and outcomes in intervention group and control group patients

	Intervention group	Control group	T-value	Sig.
	Mean (S.D.)	Mean (S.D.)		
General overuse	12.42 (2.24)	12.74 (2.63)	0.966	0.335
General harm	11.16 (2.50)	10.88 (2.96)	0.763	0.446
Beneficial effect of pharmaceuticals	14.69 (1.97)	14.96 (2.14)	0.984	0.326
Beliefs about medicines	38.27 (3.92)	38.58 (4.48)	0.545	0.587
Sensitive	15.72 (2.66)	15.55 (3.13)	0.441	0.660
Necessity beliefs	18.19 (3.68)	18.30 (4.19)	0.205	0.838
Concern beliefs	13.07 (3.36)	13.38 (4.04)	0.616	0.538
Treatment Satisfaction Questionnaire for Medication (TSQM)	83.20 (11.42)	82.54 (13.41)	0.396	0.692
Estimated weights for EQ-5D	0.63 (0.37)	0.65 (0.38)	0.414	0.680
Montgomery–Åsberg Depression Scale	22.90 (13.27)	21.44 (12.89)	0.830	0.408
Medication Adherence Scale	5.13 (1.80)	5.19 (2.05)	0.218	0.827

T-test for the difference in the characteristics of the study, according to the level of adherence

After 3 months, IG patients had significantly higher scores than CG patients for beliefs about medicine, treatment satisfaction, and medication adherence (Table 3).

**Table 3:** Clinical characteristics and outcomes in intervention group and control group patients after 3 months

	Intervention group	Control group	T-value	Sig.
	Mean (S.D.)	Mean (S.D.)		
General overuse	12.17 (2.19)	12.69 (2.62)	1.583	0.115
General harm	10.61 (2.37)	10.77 (2.93)	0.467	0.641
Beneficial effect of pharmaceuticals	14.91 (1.99)	15.11 (2.15)	0.708	0.480
Beliefs about medicines	36.84 (3.77)	38.43 (4.46)	2.855	0.005^a^
Sensitive	15.40 (2.61)	15.47 (3.11)	0.165	0.869
Necessity beliefs	18.73 (3.78)	18.46 (4.19)	0.509	0.611
Concern beliefs	12.42 (3.18)	13.25 (4.00)	1.694	0.092
Treatment Satisfaction Questionnaire for Medication (TSQM)	86.68 (11.08)	82.82 (13.40)	2.326	0.021^a^
Estimated weights for EQ-5D	0.66 (0.39)	0.65 (0.38)	0.076	0.939
Montgomery–Åsberg Depression Scale	21.07 (12.21)	21.01 (12.63)	0.036	0.971
Medication Adherence Scale	5.79 (1.89)	5.04 (1.98)	2.880	0.004^a^

^a^Significantly different

After 6 months, compared with CG patients, IG patients showed significant differences in adherence to medication, treatment satisfaction, general overuse beliefs, specific concern beliefs, and total general beliefs about medicines (Table 4). Furthermore, severity of depression and HRQoL were not significantly change between IG and CG at the end of six months. Using T-test: to test the difference between the reading at baseline & end of six month among participant in IG, a significantly improved at the end of 6 months in severity of depression 20.65 (11.97) T-value 18.09, and HRQoL 88.71 (10.77) T-value 25.53.

**Table 4:** Clinical characteristics and outcomes in intervention group and control group patients after 6 months

	Intervention group	Control group	T-value	Sig.
	Mean (S.D.)	Mean (S.D.)		
General overuse	11.93 (2.15)	12.63 (2.61)	2.197	0.029^a^
General harm	10.08 (2.25)	10.67 (2.90)	1.687	0.093
Beneficial effect of pharmaceuticals	15.35 (2.04)	15.25 (2.15)	0.356	0.722
Beliefs about medicines	35.36 (3.62)	38.12 (4.43)	5.055	<0.0001^a^
Sensitive	15.10 (2.56)	15.39 (3.10)	0.769	0.442
Necessity beliefs	19.00 (3.83)	18.50 (4.19)	0.939	0.349
Concern beliefs	12.05 (3.08)	13.14 (3.97)	2.278	0.024
Treatment Satisfaction Questionnaire for Medication (TSQM)	88.71 (10.77)	82.89 (13.40)	3.551	<0.0001^a^
Estimated weights for EQ-5D	0.68 (0.40)	0.66 (0.38)	0.356	0.722
Montgomery–Åsberg Depression Scale	20.65 (11.97)	20.86 (12.54)	0.129	0.897
Medication Adherence Scale	5.99 (1.88)	4.94 (1.94)	4.059	<0.0001^a^

^a^Significantly different

## Discussion

In this study, we examined the impact of SDM-based pharmacist interventions on patient adherence to antidepressant medications and related patient-reported outcomes. During the study, we observed changes over time in adherence, treatment satisfaction, and beliefs about antidepressants in the IG but not the CG. After 6 months, IG patients showed statistically significant increases of up to 18 % in adherence to antidepressants and 6 % in treatment satisfaction, and a decrease of 8 % in concern beliefs and general beliefs about medicines.

Our finding of improvements in adherence to antidepressants after pharmacist intervention is consistent with those of other studies (Canales, Bultman, Finley, Brook, 2005 and Rickles et al., 2005 [40, 4]). Furthermore, consistent with previous studies we did not find significant differences in the severity of depression between our groups. Most previous studies that delivered similar interventions as that described in this paper have reported no differences in the severity of depression or depressive symptoms between groups (Capoccia et al., 2004; Bosmans et al., 2007). Only one study has reported significant improvements in depressive symptoms, using the Hamilton Psychiatric Rating to measure severity of depression after pharmacist interventions in acute care psychiatric inpatients [43]. Differences in the patient sample studied (inpatient versus outpatient) may explain these differences.

However, a significant negative correlation (−0.327) between improvements in adherence and severity of depressive symptoms has been reported [44]. In this study, there were improvements in depression severity in that we observed improvements in adherence between baseline and 6 months in IG patients. However, there were no significant differences between the IG and the CG. This lack of a significant difference in depression severity between groups could result from a lack of statistical power, because the sample was calculated to obtain a difference in the main outcome (adherence to antidepressants). This may mean the study was underpowered to detect endpoint changes in secondary outcomes, such as the severity of depression. However, in this study, we used a sensitive scale (MADRS) to detect endpoint changes in depression symptoms in response to the treatment of depression [45].

Both groups reported high treatment satisfaction rates, with IG patients showing significantly greater satisfaction than did CG patients. This finding is consistent with a direct positive correlation between adherence to antidepressants and treatment satisfaction reported elsewhere [46]. It is also similar to findings from another 20 studies reported in a recent literature review [4, 47]. In contrast, both groups reported a moderate health-related quality of life, with no significant differences between groups. HRQoL is influenced by various psychological comorbidities, with depression being one of the most important of these [48, 49]. Furthermore, to assess HRQoL we used the EQ-5D, which is known to be very specific, but which produces information that lacks sensitivity. Therefore, it may not detect small changes, especially as both groups reported moderate scores at baseline.

In this study, after 3 and 6 months we detected significantly different scores in the IG for beliefs about antidepressants compared with the CG, particularly regarding general beliefs about medicines, general overuse, and specific concern beliefs. This is consistent with results reported elsewhere (Bultman et al., 2002, Rickles et al., 2005; Aikens et al., 2008). Adherence appears to be influenced by specific-concern beliefs and overuse beliefs about antidepressant medications, which is also consistent with findings from other studies [52, 53].

There are some limitations to our study. First, the study was conducted at one site only (an outpatient clinic in a psychiatric hospital) and the intervention was applied by a limited number of hospital pharmacists. This decreases the generalisability of the results to other healthcare settings. Nonetheless, our findings are consistent with studies conducted in other settings. Second, we use subjective methods (self-report scales and questionnaires) to measure patient outcomes, which may be subject to bias. However, we selected all scales carefully, ensuring they were validated with Arabic patients [24, 32, 34, 54]. Furthermore, subjective methods appear reliable and correlate with the clinical state of psychiatric patients in clinical practice [55], and patients reporting low scores are the most likely to be truthful [53]. Finally, there is a chance that researchers who were not blind to intervention group may have biased the results found. However, as those collecting the data were blind to patient group allocation we believe this had a minimal effect on our results.

Despite these limitations, the use of randomised controlled methods is a strength of this study, ensuring equal distribution of demographic and confounding factors across groups (e.g. age, gender, other symptoms, and medical problems) [56]. Furthermore, to the best of our knowledge, this is the first study to investigate pharmacist interventions with patients with depression to enhance adherence to medications using SDM processes.

Further studies are needed to evaluate the cost effectiveness of this intervention and to compare it with usual pharmacy services. New approaches must be designed to target depression symptoms in addition to improving adherence, and to improve our understanding of medication-taking behaviour among depressed patients.

## Conclusion

Pharmacist interventions based on SDM delivered to depressed patients showed a significant positive effect on adherence, treatment satisfaction, and patients’ beliefs about antidepressants. When compared with patients provided with usual care, patients given the intervention had better scores on these outcomes. This finding highlights the important role pharmacists could play in providing direct patient care in regular pharmacy practice to improve the adherence to medications and other patient-reported outcomes.

## Abbreviations

BMQ: Beliefs about medicine questionnaire
CG: Control group
IG: Intervention group
MDD: Major depressive disorder
MMAS: Morisky medication adherence scale
OPTION: Observing patient Involvement in decision
HRQOL: Health-related quality of life
SDM: Shared decision making
TSQM: Treatment satisfaction questionnaire for medication
